# Combined Therapy against Recurrent Hemangiopericytoma: A Case Report

**DOI:** 10.3969/j.issn.2095-3941.2012.02.012

**Published:** 2012-06

**Authors:** Xiao-dong Li, Jing-ting Jiang, Chang-ping Wu

**Keywords:** hemangiopericytoma, surgical procedures, radiotherapy, chemotherapy, angiogenesis inhibitors

## Abstract

Department of Oncology, The Third Affiliated Hospital of Soochow University, Changzhou 213003, China

A patient with a seven-year history of recurrent metastatic hemangiopericytoma (HPC) was admitted. During his treatment, he received surgical resection, radiotherapy, radiofrequency hyperthermia and chemotherapy using combined doxorubicin, dacarbazin, vincristine, ginsenoside Rg3, and recombinant human endostatin. This synergistic method provides an encouraging model for treating HPC.

## Introduction

Hemangiopericytoma (HPC) is a rare tumor of uncertain malignant potential. Approximately 1000 cases of HPC have been reported to date. This disease often occurs in the lower extremities (34.4%), retroperitoneum (24.5%), and meninges as well as in the head and neck ^[^^1^^]^. HPC is rarely found in the liver in these reported cases. A case of HPC in the liver with recurrence and metastasis was reviewed in this report.

## Case Report

**Figure 1:**
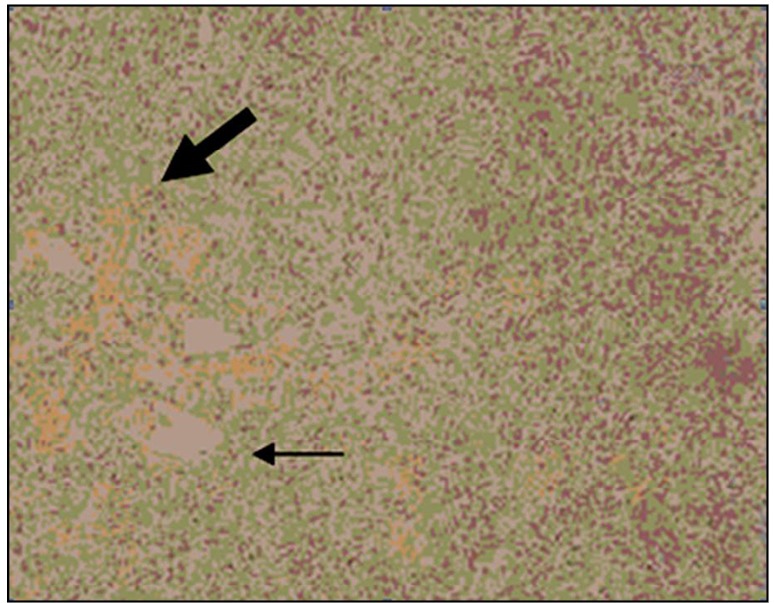
The disease was diagnosed as well-differentiated HPC. HPC are characterized histologically by spindle-shaped tumor cells (slender arrow) separated by numerous capillaries (bold arrow).

The patient was a 42-year-old man with a 7-year history of recurrent metastatic HPC. A CT scan during a physical examination in 2002 revealed a mass in the patient’s liver. He received a complete surgical resection of the mass that was pathologically and immunohistochemically confirmed as HPC (**Figure 1**). No lymph node or distant metastasis was found, and the disease was labeled as stage I (G_0_TxN_0_M_0_).

One year later, the patient was admitted because of head injury in an accident. He was found with metastasis in the brain and subsequently underwent a resection of the metastasis as well as sequential radiotherapy.

After that, residual tumors in the liver recurred twice, in 2004 and 2008, respectively, for which the patient received interventional therapy and radiation treatment.

In March 2009, a CT scan revealed a mass of 4.5 cm × 3.5 cm in the left kidney, multiple metastases in the lungs and both adrenal glands, and multiple small lymph nodes of the mediastinum. The patient underwent a complete excision of the recurrent tumor which was proved to be well-differentiated HPC. And then the patient was treated with a regimen of doxorubicin (ADM), dacarbazine (DTIC), vincristine (VCR), ginsenoside Rg3, and recombinant human endostatin (Rh endostatin) for four 21-day cycles. ADM and VCR were administered on day 1, DTIC on days 2 to 6, Rh endostatin on days 0 to 6, and ginsenoside Rg3 on days 7 to 20. The patient also received synchronous radiofrequency hyperthermia in both lung lobes during the course of treatment.

**Figure 2:**
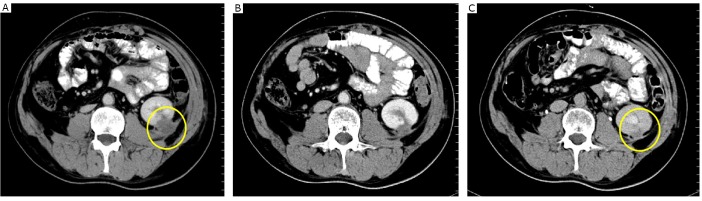
The tumor in the left kidney. A: after operation and before chemotherapy (March 2009); B: after 2 cycles of chemotherapy (May 2009); C: after 4 cycles of chemotherapy (July 2009).

**Figure 3:**
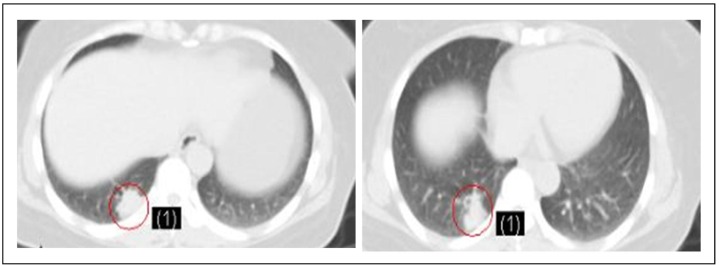
The metastatic tumor of the liver was stable after chemotherapy. The left and right pictures were taken before and after chemotherapy, respectively.

A CT scan after 2 cycles showed reduction of masses in the left kidney as well as stable lesions in the right kidney, both lungs, and liver. A CT scan after 4 cycles revealed a partial response in the left kidney (**Figure 2**) and stable disease in the right kidney, both lungs, and the liver (**Figure 3**). The disease was assessed as partially responding to the therapies.

A special symptom was noticed during the course of the disease. The patient often felt hungry which would be relieved temporarily after eating. The patient experienced no conspicuous side effects, although biochemical tests showed hypoglycemia and pigmentation of the skin, especially in the hands and feet. The patient was at a good status as of this writing.

## Discussion

A few characteristics of malignant HPC have been ignored. For instance, the tumor has typically been reported as a solitary lesion, and only a very few instances with multiple nodules have been observed ^[^^1^^]^. Another reported characteristic that is often ignored is non-islet-cell-tumor hypoglycemia (NICTH). The excessive production of glucose of the tumor during the latter phase of the disease is related to the production of the insulin-like growth factor II by the lesion ^[^^2^^]^.

This case indicated that multiple metastases in the lungs and the presence of NICTH may help diagnose HPC.

The most distinctive and consistent characteristic of HPC among sarcomas is its hypervascularity ^[^^3^^]^, providing the rationale for using anti-angiogenesis drugs, such as Rh endostatin and ginsenoside Rg3. HPC also expresses both the platelet-derived growth factor ^[^^4^^]^ and the vascular endothelial growth factor receptors ^[^^5^^]^.

Human endostatin specifically inhibits the proliferation and migration of capillary endothelial cells *in vitro* and can induce apoptosis ^[^^6^^, ^^7^^]^. Previous studies have indicated that endostatin affects a network of potentially intersecting pathways that are important in the angiogenic phenotype. And ginsenoside Rg3 is a chemical trace component (MW 784.30 Da) of Panax ginseng ^[^^8^^]^. Many studies have shown that ginsenoside Rg3 can inhibit proliferation, infiltration, and metastasis of tumor cells ^[^^9^^]^. The chemical also presents anti-angiogenesis capable of controlling some tumors ^[^^10^^]^. Ginsenoside Rg3 has been proven useful in treating lung cancer in the Chinese version of National Comprehensive Cancer Network (NCCN) guidelines.

This case presented an encouraging synergistic treatment model with combination of several disciplines: surgery, radiation, intervention, anti-angiogenesis, and chemotherapy. The regimen of VCR/ADM/DTIC combined with the two anti-angiogenic drugs produced a marked response in terms of cancer cell inhibition. However, the present case cannot prove that the combined use of ginsenoside Rg3 and endostatin is necessarily effective.

The present case is the first report wherein anti-angiogenic drugs are utilized for HPC treatment. This synergistic method actually provides an encouraging model for treating HPC although the effectiveness of single therapeutic modality remains uncertain.
